# The development of a polypoid intrapulmonary bronchogenic cyst in the bronchial lumen

**DOI:** 10.1002/rcr2.350

**Published:** 2018-07-17

**Authors:** Takamasa Hotta, Sun Rong, Yukari Tsubata, Yasuyuki Taooka, Noriaki Kurimoto, Takeshi Isobe

**Affiliations:** ^1^ Department of Internal Medicine, Division of Clinical Oncology and Respiratory Medicine Shimane University Faculty of Medicine Shimane Japan; ^2^ Department of General Medicine Aki‐Ohta Hospital Hiroshima Japan

**Keywords:** Bronchial lumen, bronchogenic cyst, polypectomy

## Abstract

Intrapulmonary bronchogenic cysts are rare in adults and most are outside the trachea and bronchi. There are reports of clear link with the trachea, but the cyst itself occurs outside the trachea. Thus, bronchoscopy will not reveal the cause, which often leads to a diagnosis by surgical resection. We herein report an extremely rare case of an intrapulmonary bronchogenic cyst that was located entirely within the left main bronchial lumen. Bronchoscopy revealed a shiny and smooth surface mass with abundant blood vessels in the lumen that blocked the left main bronchus. The patient was successfully treated with bronchoscopic resection and remained stable at 16 months of follow‐up. To our knowledge, this is the first reported case of an intrapulmonary bronchogenic cyst located entirely within the bronchial lumen.

## Introduction

Polypoid bronchogenic cysts are rare congenital bronchopulmonary malformations, commonly occurring in the mediastinum or occasionally in the intrapulmonary region. Intrapulmonary bronchogenic cysts are usually found in the lower lobes 1. When there is a possibility of link with the respiratory tract, which may cause pulmonary infection, surgical resection is recommended. Herein, we report the case of polypoid intrapulmonary bronchogenic cyst successfully treated by bronchoscopic resection alone.

## Case Report

**Figure 1:**
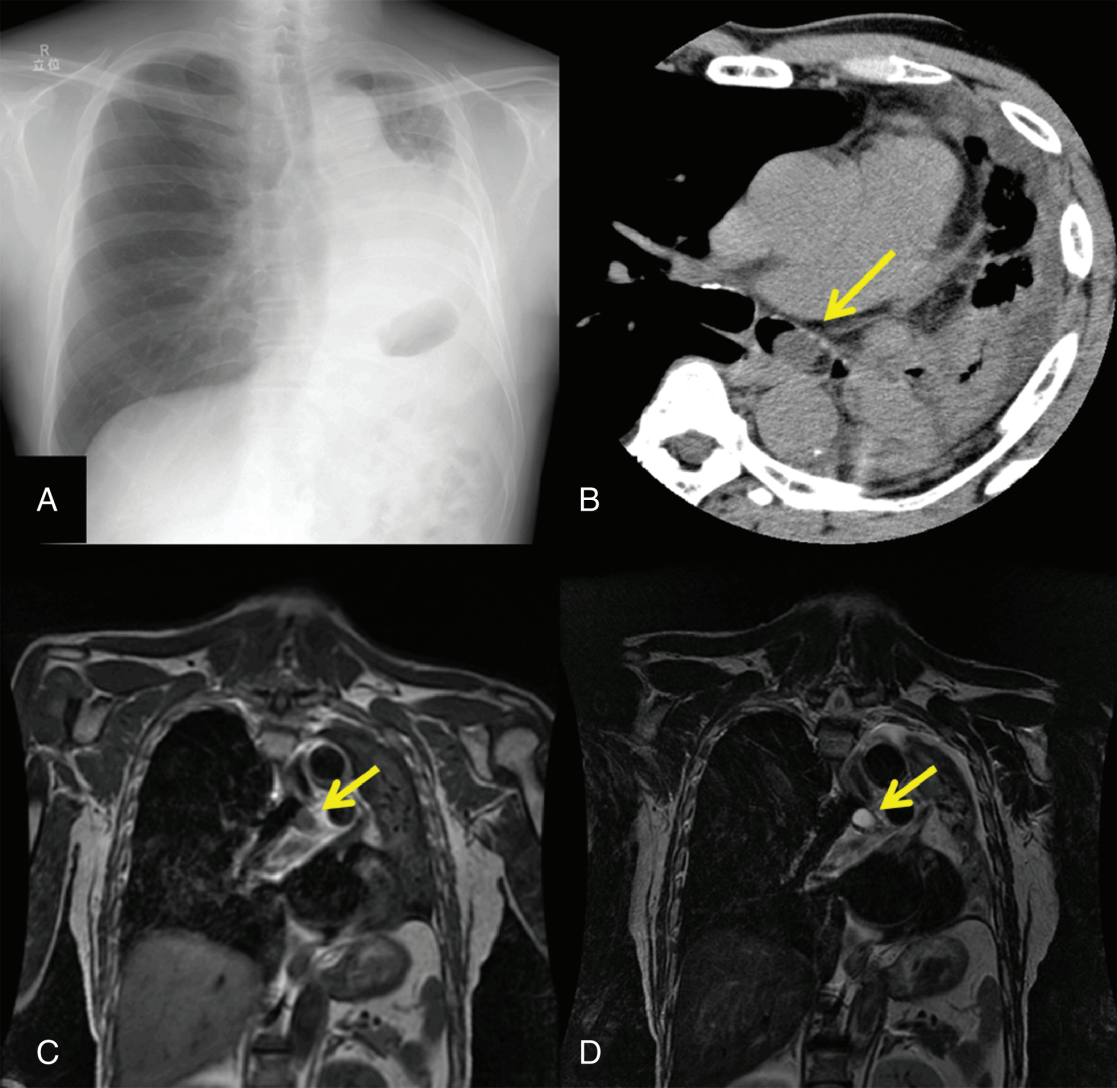
(A) The chest radiography findings led to a diagnosis of obstructive pneumonia and atelectasis. (B) Computed tomography revealed a mass in the left main bronchus. Magnetic resonance imaging revealed water signal intensity. (C) T1‐weighted imaging. (D) T2‐weighted imaging.

The patient was a 70‐year‐old man who presented with exertional dyspnea, which had persisted for one week and aggravated dyspnea, which had persisted for two weeks. He had been diagnosed with asthma two years previously and had regularly used inhaled corticosteroids since then. He had a 48 pack‐year smoking history. On admission the patient had decreased breath sounds and coarse crackles in the left lower lung field. A laboratory test showed a normal blood leukocyte count and abnormal partial pressure of oxygen (59%) and oxygen saturation (88%) values. Chest radiography revealed an area of high density in the left lower lung, which led to a diagnosis of obstructive pneumonia and atelectasis (Fig. 1A). Computed tomography (CT) revealed a mass of 20‐mm in diameter with smooth edges and a uniform density in the left main bronchus (Fig. 1B). Magnetic resonance imaging (MRI) revealed water signal intensity with low signal intensity on T1‐weighted imaging (Fig. 1C) and high signal intensity on T2‐weighted imaging (Fig. 1D), further suggesting a cystic lesion.

**Figure 2:**
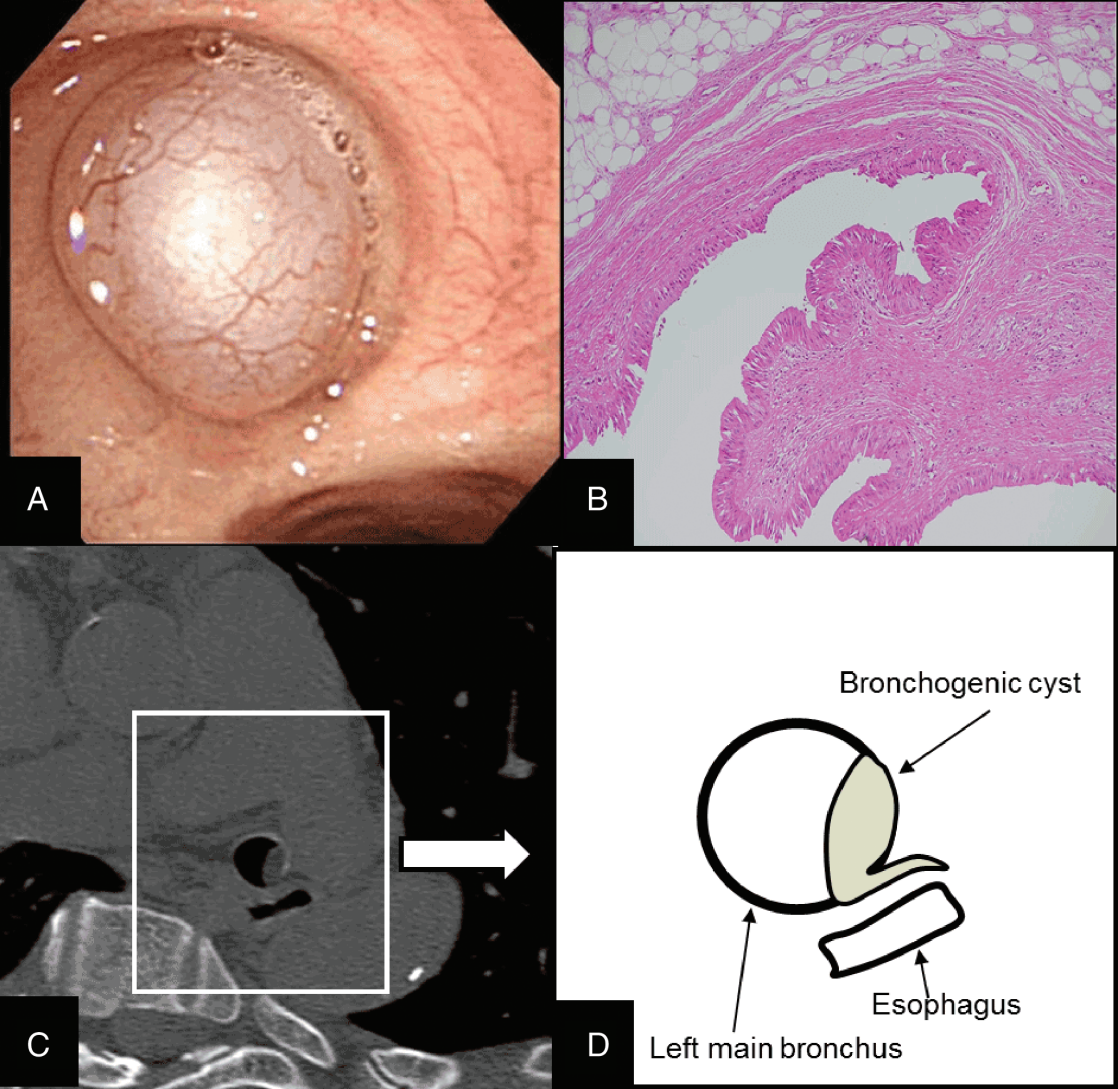
(A) Bronchoscopic view of the cyst. (B) Histology of the cystic lesion showing ciliated columnar epithelium lining cells covering the lumen. Computed tomography revealed the remaining part entering the gap between the oesophagus and the left main bronchus. (C) Axial section. (D) Schematic illustration.

Flexible bronchoscopy revealed a shiny mass with a smooth surface and abundant blood vessels blocking the left main bronchus (Fig. 2A). When grasped by forceps, the cyst wall was easily broken and a sticky transparent secretion flowed out of the cyst. Polypectomy through bronchoscopy was finally performed. Histopathology confirmed a cystic lesion that consisted of ciliated columnar epithelium lining cells covering the lumen and collagenous fibres with a bronchial gland covering the wall (Fig. 2B). The cyst was not infected and nor malignant. The patient underwent follow‐up examinations at six and 16 months after polypectomy. The remaining part did not grow in size. The detailed examination of the lesion by CT showed the oesophagus running on the dorsal side of the rear wall of the left main bronchus, which was the site of occurrence. We understood that the lesion was entering the gap between the two. This finding was suggestive of Foregut’s migration disorder (Fig. 2C, D).

## Discussion

Intrapulmonary bronchial cysts, which account for 12–23% of reported bronchogenic cysts, are rare 2, 3. The intrapulmonary bronchogenic cyst described in the present was located entirely within the left main bronchial lumen. To our knowledge, no other such cases have been reported. In the only other report describing the development of a cyst in the bronchial lumen, the main seat of the lesion was located outside the bronchus (in the lung), and only part of the cyst protruded into the bronchial lumen 4.

The clinical symptoms of bronchogenic cysts are diverse and are related to their size and the degree of organ compression. When the cyst is located entirely within the main bronchial lumen, it is easy for the severity of the symptoms to increase. In the present case, the cyst grew in size and blocked the large airways, causing symptoms similar to bronchial asthma, but without infection, which is different from ordinary cases.

MRI is very useful for the diagnosis of bronchial cysts. They are characterized by a very high and uniform signal intensity on T2‐weighted imaging 5. Although we strongly suspected a cyst, there were no published reports describing the development of a cyst in the tracheal lumen. The differential diagnoses including mucoepidermoid carcinoma, adenoid cystic carcinoma, carcinoid, cystic teratoma, intrabronchial hamartoma, inflammatory polyps and solitary papilloma. These alternatives were partly different from those of other types of bronchogenic cysts because of the unique location.

In conclusion, intrapulmonary bronchogenic cysts may occur in the bronchial lumen. Bronchoscopic treatment may be effective in such cases.

### Disclosure Statement

Appropriate written informed consent was obtained for publication of this case report and accompanying images.
